# A phase Ib study to assess the efficacy and safety of vismodegib in combination with ruxolitinib in patients with intermediate- or high-risk myelofibrosis

**DOI:** 10.1186/s13045-018-0661-x

**Published:** 2018-09-24

**Authors:** Stephen Couban, Giulia Benevolo, William Donnellan, Jennifer Cultrera, Steffen Koschmieder, Srdan Verstovsek, Gregory Hooper, Christian Hertig, Maneesh Tandon, Natalie Dimier, Vikram Malhi, Francesco Passamonti

**Affiliations:** 1 Queen Elizabeth II Health Sciences Centre, 1278 Tower Road, Room 420, Halifax, Nova Scotia B3H 2V7 Canada; 2 Azienda Ospedaliero-Universitaria Città della Salute e della Scienza di Torino, San Giovanni Battista, Corso Bramante 88/90, 10126, Torino, Italy; 3 Sarah Cannon Research Institute, 250 25th Ave North, Suite 412, Nashville, TN 37203 USA; 4 Florida Cancer Specialists, 1400 North US Highway 441, Suite 540, The Villages, FL 32159 USA; 5 Department of Hematology, Oncology, Hemostaseology, and SCT, Faculty of Medicine, Rheinisch-Westfälische Technische Hochschule Aachen University, Pauwelsstrasse 30, 52074 Aachen, Germany; 6 Department of Leukemia, The University of Texas MD Anderson Cancer Center, 1515 Holcomb Blvd., Unit 428, Houston, TX 77030 USA; 7 Roche Products Ltd, 6 Falcon Way, Shire Park, Welwyn Garden City, AL7 1TW UK; 8 Roche Clinical Science, F. Hoffmann-La Roche Ltd., Bldg. 001, Room 07.S373, CH-4070 Basel, Switzerland; 9 Genentech Research and Early Development, Genentech, Inc., 1 DNA Way, South San Francisco, CA 94080 USA; 10 Università degli Studi dell’Insubria, Viale Luigi Borri, 57, 21100 Varese, VA Italy; 11 Queen Elizabeth II Health Sciences Centre, Room 430, Bethune Building, VG Site, 126 South Park Street, Halifax, Nova Scotia B3H 2V9 Canada

**Keywords:** Vismodegib, Ruxolitinib, Myelofibrosis, Hedgehog pathway inhibitor, Hematopoiesis, Hematologic malignances

## Abstract

**Background:**

The JAK inhibitor (JAKi) ruxolitinib is standard treatment for myelofibrosis (MF), but some patients are unresponsive. Pre-clinical and clinical data suggest that addition of a Hedgehog pathway inhibitor (HPI) to ruxolitinib might improve response. Vismodegib is an HPI approved for treatment of locally advanced and metastatic basal cell carcinoma. The MYLIE study assessed the safety and efficacy of combining ruxolitinib with vismodegib in ruxolitinib-naive patients with MF and characterized the pharmacokinetics (PK) of vismodegib in this setting.

**Methods:**

In this phase Ib study, ten patients with intermediate- or high-risk primary or secondary MF received open-label vismodegib (150 mg/day orally) and ruxolitinib (15 or 20 mg orally twice daily, depending on baseline platelet count) for up to 48 weeks, or until withdrawal or discontinuation. PK samples were collected throughout the study for comparison with other patient populations. Efficacy outcomes at week 24 included spleen response (≥ 35% reduction in volume by imaging) and improvement in bone marrow fibrosis by central and investigator assessment, symptom response (≥ 50% reduction in Myeloproliferative Neoplasm Symptom Assessment Form Total Symptom score), and anemia response (per International Working Group for Myeloproliferative Neoplasms Research and Treatment revised response criteria).

**Results:**

As of November 17, 2017, eight patients had completed 48 weeks of treatment with vismodegib and ruxolitinib; two discontinued treatment early. At week 24 (± 1 week), three patients experienced a spleen response by central review and no patients showed a 1-grade improvement in bone marrow fibrosis by central review. Five patients experienced symptom response at week 24, and no patients experienced an anemia response. The most common adverse events were muscle spasm (100% of patients), alopecia (70%), dysgeusia (50%), thrombocytopenia (50%), and nausea (40%); these events were predominantly grade 1/2. Three patients experienced a total of six serious adverse events.

**Conclusions:**

The combination of vismodegib and ruxolitinib was tolerable and no new safety signals were seen, but there was no evidence that the addition of vismodegib to ruxolitinib improved any of the efficacy outcome measures assessed. Further evaluation of this combination will not be pursued.

**Trial registration:**

ClinicalTrials.gov, NCT02593760. Registered November 2, 2015.

**Electronic supplementary material:**

The online version of this article (10.1186/s13045-018-0661-x) contains supplementary material, which is available to authorized users.

The JAK inhibitor (JAKi) ruxolitinib is standard treatment for intermediate- or high-risk myelofibrosis (MF), but fewer than 50% of patients achieve spleen response (≥ 35% reduction in spleen volume) or significant improvement in symptom burden [1, 2].

The Hedgehog signaling pathway is involved in primitive and definitive bone marrow hematopoiesis and maintenance of hematopoietic precursors [3]. Pre-clinical and clinical data suggest that addition of a Hedgehog pathway inhibitor (HPI) to ruxolitinib may improve response [4, 6]. The HPI vismodegib is approved for the treatment of advanced basal cell carcinoma [5].

We present findings from MYLIE, an open-label, multi-center, phase Ib study (ClinicalTrials.gov, NCT02593760) of vismodegib plus ruxolitinib in patients with intermediate- or high-risk MF.

Eligible patients were adults with pathologically confirmed primary MF, post-polycythemia vera MF, or post-essential thrombocythemia MF who were considered intermediate-1, intermediate-2, or high risk, with a peripheral blood blast count < 10% and palpable splenomegaly > 5 cm below the left costal margin, and who had no prior treatment with a JAKi or an HPI.

Patients received vismodegib (150 mg/day orally) and ruxolitinib (starting dose 15 or 20 mg orally twice daily, depending on baseline platelet count) for up to 48 weeks, or until disease progression, unacceptable toxicity, or consent withdrawal.

Efficacy end points were assessed at 24 and 48 weeks after the first dose of study medication and included spleen response rate (≥ 35% reduction in spleen volume), disease response rate (complete remission [CR] and partial remission [PR]), clinical improvement (anemia, spleen, or symptom response without disease progression or increase in severity of anemia, thrombocytopenia, or neutropenia), anemia response rate, and improvement in bone marrow fibrosis of ≥ 1 grade (Additional file 1). Pharmacokinetics of total and unbound vismodegib were characterized using pre-dose samples obtained at weeks 6, 12, 24, 36, and 48 and were compared with data from patients with advanced basal cell carcinoma [7–9].

Ten patients were enrolled at four sites in the USA (2), Canada (1), and Italy (1) (Table 1). Patients received vismodegib plus ruxolitinib for a median of 330.0 days. Eight patients completed 48 weeks of treatment; one patient discontinued owing to lack of efficacy at 35 weeks and one because of a vismodegib-related adverse event (AE; dysgeusia) at 23 weeks.

**Table 1:** Baseline characteristics

	*N* = 10
Age, median (range), years	67.5 (46–82)
Sex, *n*	
Male	7
Female	3
ECOG performance status, *n*	
0	4
1	6
Myelofibrosis type, *n*	
Primary MF	6
Post-ET	3
Post-PV	1
DIPSS risk status, *n*	
High	1
Intermediate-2	6
Intermediate-1	3
Transfusion-dependent at study entry	
Yes	3
No	7

*DIPSS* International Working Group-Myeloproliferative Neoplasms Research and Treatment Dynamic International Prognostic Scoring System, *ECOG* Eastern Cooperative Oncology Group performance status, *PV* polycythemia vera

At week 24, spleen response was observed in one and three patients by investigator and central review, respectively (Fig. 1a). At week 48, of eight patients, two and four patients had spleen response by investigator and central review, respectively.

**Fig. 1:**
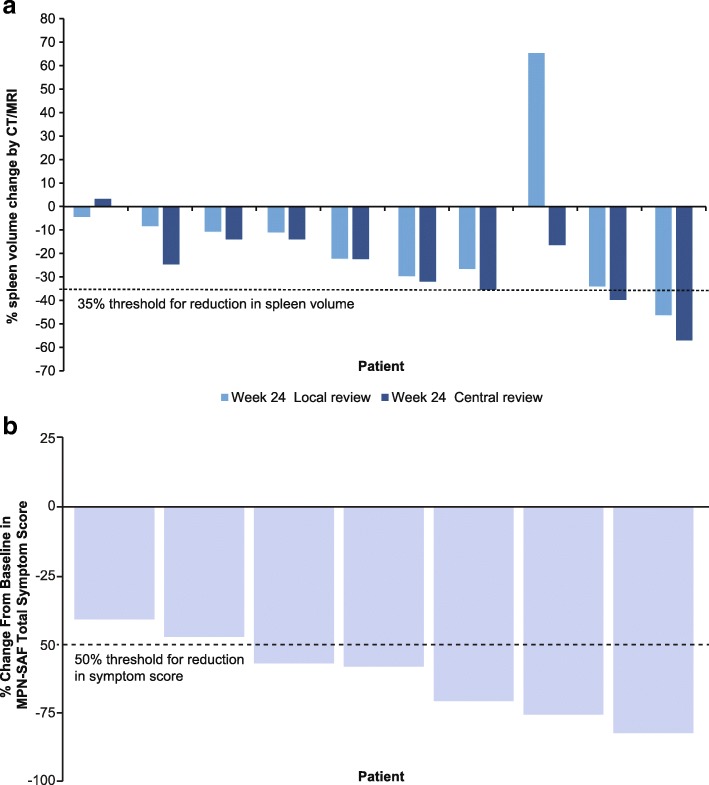
Spleen volume change from baseline per central and investigator review (**a**) and symptom response change (**b**) at week 24, by patient. Threshold for clinical efficacy at 35% reduction in spleen volume and 50% reduction in the MPN-SAF TSS is indicated by the dotted line. Spleen volume was assessed by CT or MRI, both by a local radiologist and a central independent review committee. Symptom score data were not available for three patients, including the patient who discontinued early. *CT* computed tomography; *MPN-SAF TSS* Myeloproliferative Neoplasm Symptom Assessment Form Total Symptom Score; *MRI* magnetic resonance imaging

Of seven evaluable patients, five had symptom response at week 24 (Fig. 1b). At week 48, symptom response was achieved in two patients with no previous symptom response, three patients maintained symptom response from week 24, and two were not evaluable. No patients experienced anemia response. Of nine patients evaluable for disease response at week 24, one experienced PR, one experienced clinical improvement, and seven had stable disease. Of eight patients on study at week 48, one maintained clinical improvement, six had stable disease, and the patient with PR had relapsed.

From baseline, no patients experienced a ≥ 1-grade improvement in fibrosis at week 24 per central review (Additional file 1: Table S1). Of five patients with bone marrow biopsy specimens at week 48, a 1-grade improvement in fibrosis was observed in one and two patients by central review and local pathology review, respectively.

All ten patients experienced at least one AE (Additional file 1: Table S2): five patients (50%) experienced grade ≥ 3 AEs, and three patients (30%) experienced a total of six serious AEs (grade 4 anemia, grade 3 pneumonia, grade 4 sepsis, grade 3 vomiting, grade 3 lung infiltration, grade 3 infectious enterocolitis). No patients died during the study period. Dose interruptions or modifications are shown in Additional file 1: Table S3.

Total and unbound steady-state vismodegib plasma concentrations in patients with MF were consistent with those obtained in patients with basal cell carcinoma (Additional file 1: Table S4) [7, 8].

Vismodegib combined with ruxolitinib did not result in greater efficacy than ruxolitinib alone [1], consistent with other trials of HPIs in MF [6, 10]. The combination was tolerable, and no new safety signals were seen. However, because of lack of evidence of a disease-modifying effect after addition of vismodegib to ruxolitinib, further evaluation of this combination will not be pursued.

## Additional file

Additional file 1: **Supplementary methods. Table S1.** Bone marrow fibrosis grade. **Table S2.** Treatment-emergent AEs of any grade that occurred in ≥ 2 patients. **Table S3.** AEs leading to treatment interruption or modification. **Table S4.** Mean (± SD) total and unbound steady-state vismodegib plasma concentration. (DOCX 24 kb)

## Abbreviations

AE: Adverse event
CR: Complete remission
CT: Computed tomography
DIPSS: International Working Group-Myeloproliferative Neoplasms Research and Treatment Dynamic International Prognostic Scoring System
ECOG: Eastern Cooperative Oncology Group performance status
HPI: Hedgehog pathway inhibitor
JAK: Janus kinase
JAKi: JAK inhibitor
MF: Myelofibrosis
MPN-SAF: Myeloproliferative Neoplasm Symptom Assessment Form
MRI: Magnetic resonance imaging
PK: Pharmacokinetics
PR: Partial remission
PV: Polycythemia vera
SAE: Serious adverse event
TSS: Total symptom score

## References

[1] Verstovsek S, Mesa RA, Gotlib J, Levy RS, Gupta V, DiPersio JF (2012). A double-blind, placebo-controlled trial of ruxolitinib for myelofibrosis. N Engl J Med.

[2] Harrison C, Kiladjian J-J, Al-Ali HK, Gisslinger H, Waltzman R, Stalbovskaya V (2012). JAK inhibition with ruxolitinib versus best available therapy for myelofibrosis. N Engl J Med.

[3] Tibes R, Mesa RA (2014). Targeting hedgehog signaling in myelofibrosis and other hematologic malignancies. J Hematol Oncol.

[4] Bhagwat N, Keller M, Rampal R, Koppikar P, Shank K, De Stanchina E (2013). Improved efficacy of combination of JAK2 and hedgehog inhibitors in myelofibrosis [abstract]. Blood.

[5] AMA 10th. Erivedge (vismodegib) [package insert]. South San Francisco: Genentech USA, Inc.; 2017.

[6] Gupta V, Harrison CN, Hasselbalch H, Pieri L, Koschmieder S, Cervantes F (2015). Phase 1b/2 study of the efficacy and safety of sonidegib (LDE225) in combination with ruxolitinib (INC424) in patients with myelofibrosis [abstract]. Blood.

[7] Sekulic A, Migden MR, Oro AE, Dirix L, Lewis KD, Hainsworth JD (2012). Efficacy and safety of vismodegib in advanced basal-cell carcinoma. N Engl J Med.

[8] Basset-Seguin N, Hauschild A, Kunstfeld R, Grob J, Dreno B, Mortier L (2017). Vismodegib in patients with advanced basal cell carcinoma: primary analysis of STEVIE, an international, open-label trial. Eur J Cancer.

[9] Couban S, Benevolo G, Donnellan W, Cultrera J, Koschmieder S, Verstovsek S (2017). Phase 1b results of a study to assess the efficacy and safety of vismodegib in combination with ruxolitinib in patients with intermediate- or high-risk myelofibrosis. Blood.

[10] Sasaki K, Gotlib JR, Mesa RA, Newberry KJ, Ravandi F, Cortes JE (2015). Phase II evaluation of IPI-926, an oral Hedgehog inhibitor, in patients with myelofibrosis. Leuk Lymphoma.

