# Supplementary material for: A phase Ib study to assess the efficacy and safety of vismodegib in combination with ruxolitinib in patients with intermediate- or high-risk myelofibrosis
Source: J Hematol Oncol. 2018 Sep 24;11:122. doi: 10.1186/s13045-018-0661-x (PMC6154811; doi:10.1186/s13045-018-0661-x)
Supplement: Supplementary file 1 — Supplementary methods. Table S1. Bone marrow fibrosis grade. Table S2. Treatment-emergent AEs of any grade that occurred in ≥ 2 patients. Table S3. AEs leading to treatment interruption or modification. Table S4. Mean (± SD) total and unbound steady-state vismodegib plasma concentration. (DOCX 24 kb) [file 13045_2018_661_MOESM1_ESM.docx]

**Supplemental Information**

**Supplementary Methods**

Spleen volume and bone marrow fibrosis were assessed by local imaging and pathology, respectively, and by independent review per International Working Group-Myeloproliferative Neoplasms Research and Treatment (IWG-MRT) revised response criteria. In addition, spleen size was assessed by palpation by the investigator at screening and scheduled postbaseline disease assessments. Symptom response was assessed by patients using the Myeloproliferative Neoplasm Symptom Assessment Form (MPN-SAF); response was defined as a ≥ 50% reduction in total symptom score (TSS). Improvement in bone marrow fibrosis was assessed using the European consensus grading system. Safety was evaluated using National Cancer Institute Common Terminology Criteria for Adverse Events, version 4.0.

**Table S1** Bone marrow fibrosis grade

| **Patient (review)** | **Baseline** | **Week 24** | **Week 36** | **Week 48** | **Last result compared**  **with baseline** |
| --- | --- | --- | --- | --- | --- |
| 1 (Central) | MF-3 | MF-3 | MF-3 | MF-3 | No change |
| 1 (Local) | MF-3 | MF-3 | MF-3 | MF-3 | No change |
| 2 (Central) | MF-1 | MF-1 | MF-1 | ND | No change |
| 2 (Local) | MF-1 | NR | MF-1 | ND | No change |
| 3 (Central) | MF-2 | MF-2 | MF-3 | MF-3 | Worse |
| 3 (Local) | MF-3 | MF-2 | MF-3 | MF-2 | Improved |
| 4 (Central) | MF-3 | MF-3 | MF-3 | ND | No change |
| 4 (Local) | MF-3 | MF-3 | MF-3 | ND | No change |
| 5 (Central) | MF-1 | MF-1 | MF-2 | MF-1 | No change |
| 5 (Local) | MF-2 | MF-2 | MF-1 | MF-1 | Improved |
| 6 (Central) | MF-2 | MF-3 | MF-3 | MF-3 | Worse |
| 6 (Local) | MF-2 | MF-3 | MF-3 | MF-3 | Worse |
| 7 (Central) | MF-2 | MF-2 | MF-2 | MF-2 | No change |
| 7 (Local) | MF-2 | MF-2 | MF-2 | MF-2 | No change |
| 8^a^ (Central) | MF-2 | – | – | – | NA |
| 8^a^ (Local) | MF-2 | – | – | – | NA |
| 9 (Central) | MF-2 | MF-2 | MF-2 | MF-1 | Improved |
| 9 (Local) | MF-1 | MF-1 | ND | ND | No change |
| 10 (Central) | MF-3 | MF-3 | MF-3 | – | No change |
| 10 (Local) | MF-2 | NR | MF-3 | – | Worse |

*MF* myelofibrosis, *NA* not applicable, *ND* not done, *NR* not recorded, – no data because the patient discontinued treatment

Grades were assessed centrally according to the European Consensus on Grading of Bone Marrow Fibrosis.

^a^This patient discontinued early because of an adverse event and did not undergo bone marrow biopsy.

**Table S2** Treatment-emergent AEs of any grade that occurred in ≥ 2 patients

| **MedDRA preferred term, *n*** | **Any grade** | **Grade 3/4** |
| --- | --- | --- |
| Muscle spasm | 10 | – |
| Alopecia | 7 | – |
| Dysgeusia | 5 | – |
| Thrombocytopenia | 5 | 2 (grade 3) |
| Nausea | 4 | – |
| Anemia | 3 | 2 (grade 3)  1 (grade 4) |
| Abdominal pain | 3 | – |
| *Clostridium difficile* infection | 2 | 1 (grade 3) |
| Fall | 2 | – |
| Dyspnea | 2 | – |
| Oropharyngeal pain | 2 | – |
| Pyrexia | 2 | – |

*AE* adverse event, *MedDRA* Medical Dictionary for Regulatory Activities

For frequency counts by preferred term, multiple occurrences of the same AE in an individual were counted only once.

**Table S3** AEs leading to treatment interruption or modification

| **Patient** | **Vismodegib interruption^a^** | **Ruxolitinib interruption/modification** |
| --- | --- | --- |
| 1 | – | Thrombocytopenia |
| 2 | – | – |
| 3 | Vomiting (SAE) | Anemia (SAE) /neutropenia/thrombocytopenia  Vomiting (SAE) |
| 4 | – | Platelet count decrease |
| 5 | – | Thrombocytopenia |
| 6 | – | Thrombocytopenia |
| 7 | – | Infectious enterocolitis (SAE) |
| 8 | Dysgeusia^b^ | Dysgeusia |
| 9 | Sepsis (SAE) and lung infiltration^c^ (SAE) | Thrombocytopenia  Sepsis (SAE) and lung infiltration (SAE) |
| 10 | Dysgeusia and muscle spasm | – |

*AE* adverse event, *SAE* serious adverse event, – no AE

^a^Per protocol, modification of vismodegib dose was not allowed.

^b^Patient did not restart vismodegib after interruption and discontinued study treatment because the interruption was >4 weeks, at which point the protocol required discontinuation of study treatment.

^c^Patient experienced two separate interruptions of vismodegib: one because of sepsis and one because of lung infiltration.

**Table S4** Mean (±SD) total and unbound steady-state vismodegib plasma concentration

| **Patient data** | **Total vismodegib, µM** | **Unbound vismodegib, µM** |
| --- | --- | --- |
| MYLIE week 6 (*n* = 10) | 20.3 ± 7.9 | 0.261 ± 0.183 |
| MYLIE week 12 (*n* = 9) | 25.2 ± 10.3 | 0.266 ± 0.154 |
| MYLIE week 24 (*n* = 9) | 27.4 ± 8.4 | 0.290 ± 0.150 |
| MYLIE week 36 (*n* = 8) | 25.5 ± 9.1 | 0.346 ± 0.145 |
| MYLIE week 48 (*n* = 8) | 24.5 ± 5.9 | 0.321 ± 0.099 |
| **Average across all timepoints in MYLIE (*n* = 44)^a^** | **24.5 ± 8.45** | **0.290 ± 0.149** |
| ERIVANCE^1^ (*n* = 78) | 27.0 ± 9.7 | ND |
| STEVIE^2^ (*n* = 44) | 22.7 ± 8.5 | 0.242 ± 0.116 |

*ND* not determined, *PK* pharmacokinetics, *SD* standard deviation

^a^*n* is the total number of observations used to calculate the average.

**Supplementary References**

1. Sekulic A, Migden MR, Oro AE, Dirix L, Lewis KD, Hainsworth JD, et al. Efficacy and safety of vismodegib in advanced basal-cell carcinoma. N Engl J Med. 2012;366(23):2171–9.
2. Basset-Seguin N, Hauschild A, Kunstfeld R, Grob J, Dreno B, Mortier L, et al. Vismodegib in patients with advanced basal cell carcinoma: primary analysis of STEVIE, an international, open-label trial. Eur J Cancer. 2017;86:334–48.
